# The Impact of Adverse Childhood Experiences on Mobile Phone Addiction in Chinese College Students: A Serial Multiple Mediator Model

**DOI:** 10.3389/fpsyg.2020.00834

**Published:** 2020-05-13

**Authors:** Wenfu Li, Xueting Zhang, Minghui Chu, Gongying Li

**Affiliations:** School of Mental Health, Jining Medical University, Jining, China

**Keywords:** adverse childhood experience, mobile phone addiction, attachment anxiety, attachment avoidance, interpersonal relationship, multiple mediating model

## Abstract

Mobile phone addiction is a universal phenomenon that has attracted a lot of attention in recent years. Previous researches revealed a significant relation between adverse childhood experiences (ACEs) and addiction. This study further investigated the association between ACEs and mobile phone addiction, and the mediating effects of attachment styles and interpersonal relationships. The cross-sectional design and multiple questionnaires, namely, the Revised Adverse Childhood Experience Questionnaire, the Mobile Phone Addiction Index, the Revised Adult Attachment Scale (AAS), and the Interpersonal Relationship Comprehensive Diagnostic Scale (IRCDS) were used in the sample of 345 university students. Correlation analysis revealed that adverse childhood experience, attachment anxiety, attachment avoidance, interpersonal relationship, and mobile phone addiction were significantly positively correlated with each other. Results of regression analysis showed that attachment style and interpersonal relationship played multiple mediation roles in the association between adverse childhood experience and mobile phone addiction. That is, (1) adverse childhood experience was positively related to mobile phone addiction, (2) both attachment anxiety and interpersonal relationship played partial and parallel mediating roles between adverse childhood experience and mobile phone addiction, and (3) attachment anxiety/avoidance and interpersonal relationship mediated the relationship between adverse childhood experience and mobile phone addiction sequentially. These results indicated that mobile phone addiction among college students who had adverse childhood experience can be relieved by way of the remission of attachment anxiety, reduction of attachment avoidance, and improvement of interpersonal relationship.

## Introduction

Mobile phone is one of the greatest inventions in modern society and facilitates our lives in many different ways. However, the excessive reliance on smartphone, called mobile phone addiction, causes compelling questions, which attracted a lot of attention in recent years (Salehan and Negahban, 2013; Demirci et al., 2015; Aljomaa et al., 2016; Enez Darcin et al., 2016). Mobile phone addiction is a phenomenon where individuals indulged in activities based on their phone, had continued strong craving and dependency on their phone, and had social and mental function impairments (Yen et al., 2009). Intensive research demonstrated that the problematic use of mobile phone was associated with physical problems such as low sleep quality, muscular pain, and eye disease (De-Sola et al., 2017; Lee et al., 2017), and mental problems such as depression, anxiety, autism, attention deficit-hyperactivity disorder (ADHD), and low self-confidence (Elhai et al., 2016; Panova and Lleras, 2016; Brown et al., 2017; Berg et al., 2018; Thomée, 2018). Mobile phone is an important part of undergraduate life (Yen et al., 2009). Undergraduates in China use smartphone to shop online, to make payment, to play video games, to chat online, and to call others. Thus, the examination of mobile phone addiction in college students has great realistic significance.

Numerous studies revealed some predictors of mobile phone addiction such as quality of life (Kumcagiz, 2018), social self-efficacy (Kim and Koh, 2018), avoidant attachment (Kim and Koh, 2018) and self-esteem (Bianchi and Phillips, 2005). The relationship between adverse childhood experiences (ACEs) and addiction was proved by numerous studies (Sacco et al., 2007; Schellekens et al., 2013; Giordano et al., 2014; Puetz and McCrory, 2015; Levenson, 2016), but whether ACEs affect mobile phone addiction remains unclear. The present study aims to investigate the relationship between ACEs and problematic phone use and the role of adult attachment and interpersonal relationship. The relationships between pairs of the abovementioned variables are introduced below.

### ACEs and Mobile Phone Addiction

Adverse childhood experiences are generally characterized by physical/emotional neglect, physical/psychological/sexual abuse, low socioeconomic status, and loss of a close family member that happened before 18 years old (Finkelhor et al., 2015; Wang et al., 2018). Previous studies showed that ACEs were related to psychological and physiological health output (De Venter et al., 2013; Hughes et al., 2017). ACEs are considered as an efficient predicated variable of problem behaviors, such as domestic violence (Pournaghash-Tehrani and Feizabadi, 2009) and criminal activity (Fox et al., 2015). In particular, loads of studies reported a strong association between the quantity of ACEs and the risk for various types of abuse or addiction, for example, alcohol abuse (Dube et al., 2002), food addiction (Holgerson et al., 2018), tobacco dependence (Walsh and Cawthon, 2014), and drug addiction (Gomez et al., 2018). Despite the close relationship between ACE exposure and addictive behaviors, researchers have not explored the relation between ACEs and mobile phone addiction. Thus, the possible mechanism behind how ACEs influence mobile phone addiction needs to be explored further.

### Attachment Style and Mobile Phone Addiction

Attachment theory initially emphasized the significance of the mother–infant attachment relationship (Bowlby, 1969). Later attachment studies had extended beyond infant attachment to adult (Mikulincer and Shaver, 2007). Attachment theory indicated that the cognitive models that developed during a person’s childhood might potentially affect his grown-up life (Collins and Read, 1994; Mikulincer and Shaver, 2007). The styles of attachment helped describe and interpret individual behavior differences of grown-ups (Collins, 1996). Previous research indicated that various addictions were the result of attachment disorder (Khantzian, 2011). For example, Shin et al. (2011) found that attachment anxiety predicted alcohol use, while attachment anxiety and avoidance predicted problematic internet use. More problematic online gaming actions were also found on participants with attachment anxiety and avoidance styles rather than participants with secure attachment styles (Suárez et al., 2012). Recent studies further found that attachment anxiety was positively related to mobile phone addiction in Chinese college students (Han et al., 2017; Yuchang et al., 2017). Kim and Koh (2018) also found that attachment avoidance influenced mobile phone addiction through self-esteem and anxiety. The cognitive–behavioral model of Davis (2001) indicated that insecure attachment styles were a reliable predictor of some substance abuse. Addiction was regarded as attachment disorder (Parolin and Simonelli, 2016). Based on the close association between attachment and addictive behaviors, the present study assumed that attachment styles would predict the severity levels of mobile phone addiction.

### Interpersonal Relationships and Mobile Phone Addiction

Numerous studies showed that there was a close association between interpersonal relationships and addictive behaviors. A study on Korean middle school students found that the problems of interpersonal relationships was correlated positively with internet addiction (Seo et al., 2009). A research on internet game players found that the internet game addiction was positively correlated with the quality of interpersonal relations with their parents and peers (Kwon et al., 2011). A study on Chinese middle school students found that the problematic interpersonal relationships were significantly correlated with internet addiction disorder (Huang, 2009). Other studies also found that interpersonal relationships were related to Facebook addiction (Tang et al., 2016) and video game addiction (Choi et al., 2017). Based on the association between interpersonal relationship and phone addiction, internet addiction, and video game disorder (Khang et al., 2013; Hadlington, 2015), the present study assumes that weak interpersonal relationships positively related to mobile phone addiction.

### ACEs, Attachment Style, and Interpersonal Relationships

Adverse childhood experiences were related to attachment style. For example, Raby et al. (2017) found that child abuse experiences were positively correlated with insecure attachment styles in a longitudinal study. Morina et al. (2016) further found that previous adverse experiences could reduce secure attachment style and enhance avoidance of attachment seeking. Other studies also found similar association between abuse experiences and insecure attachment styles (Bakermans-Kranenburg and van Ijzendoorn, 2009; Corcoran and McNulty, 2018). Attachment theory indicated that people who have went through adverse experiences in early childhood would have difficulties in the communication with others in grown life (Frederick and Goddard, 2008). Berry et al. (2006) found that insecure attachment styles were correlated with the problem of interpersonal connection. Therefore, adverse experiences might influence interpersonal relationship through the mediated effect of attachment styles.

### A Serial Multiple Mediator Model

In the first place, based on the relationships between attachment style and mobile phone addiction (Han et al., 2017; Yuchang et al., 2017; Kim and Koh, 2018), attachment style might be conducted as a crucial variable in the association between ACEs and problematic behaviors. Studies did indicate that both attachment anxiety and avoidance mediated the link between childhood adversity and eating disorders in clinical sample (Tasca et al., 2013). Therefore, it was reasonable that the relationship between ACEs and mobile phone addiction might be mediated by the effect of attachment styles.

In addition, ACEs had a significant influence on interpersonal relationships. It was indicated that the child emotional abuse induced more interpersonal problem with their parents, teachers, and peers (Lin et al., 2016). Other research found that the child sexual abuse damaged the interpersonal functioning in a sample of 2892 young adult women. A recent study also found that the early adversity positively related to the enhanced interpersonal difficulties in adulthood in a large sample of 4006 (Poole et al., 2018). Then, it was obvious that the association between ACEs and mobile phone addiction might be mediated by interpersonal relationships.

Furthermore, attachment styles played an important role on interpersonal relationships. Berry et al. (2006) found that there was a positive association between insecure attachment styles and interpersonal problems. Koelen et al. (2016) also found that there was a close association between attachment avoidance and interpersonal problems. Therefore, attachment style was closely related to interpersonal relationships.

In conclusion, the above discussions indicated obviously that ACEs, attachment styles, interpersonal relationships, and mobile phone addiction were related to each other. In other words, the present study intended to explore the serial multiple mediating effects of attachment styles and interpersonal relationships between ACEs and mobile phone addiction in a sample of university students. First, we anticipated that the ACEs would be related to mobile phone addiction in Chinese college students. Second, we expected that the attachment styles and interpersonal relationships would mediate the association between ACEs and mobile phone addiction, respectively. In particular, it was assumed that attachment style and interpersonal relationship would play a serial mediation effect between ACE and mobile phone addiction.

## Materials and Methods

### Participants

The present research enrolled 400 undergraduate students from Jining Medical University (Shandong, China). All participants own a smartphone and often use it to go online or play video games as part of their everyday activities. All subjects signed the written informed consent form before they joined the research, which was authorized by the Institutional Human Participants Review Board of Jining Medical University. All questionnaires were filled out in a psychological measurement room. All the measures used in the present study were revised or developed using the standard procedure. All the items were in an easy-to-understand Chinese version. The trained graduate students who majored in psychology issued and recovered the written scales. They also were responsible for the explanation of possible doubt to avoid any confusion. It takes about 15 min to fill out the questionnaires. The data of 1 blank and 54 incomplete questionnaires were removed. This study included 345 participants (128 males and 217 females; mean age = 19.75 years, standard deviation = 1.32, age range from 17 to 25 years), including medical students (*n* = 221, 64.1%) and non-medical students (*n* = 124, 35.9%); birthplace in cities (*n* = 146, 42.3%) and rural areas (*n* = 199, 57.7%); and only child (*n* = 120, 34.8%) and not an only child (*n* = 225, 65.2%).

### Measures

#### Revised Adverse Childhood Experience Questionnaire (ACEQ-R)

The Chinese version (Wang et al., 2018) of ACEQ-R (Finkelhor et al., 2015) was adopted, which consisted of 14 items and assessed personal experiences like physical/emotional neglect, physical/psychological/sexual abuse, and low socioeconomic status, which happened before 18 years old. Each item meant one kind of ACE and was answered with 1 if the subjects experienced it and 0 if the subjects never experienced it. The score of ACEQ-R was equal to the number of items answered with 1. The higher the score of ACEQ-R, the more the kinds of adverse experience were. This Chinese version had satisfactory reliability and validity (Wang et al., 2018). The Cronbach’s alpha was 0.713.

#### Mobile Phone Addiction Index (MPAI)

The Chinese version (Huang et al., 2014) of MPAI (Leung, 2008) contained 17 items, which include items such as “You have attempted to spend less time on your mobile phone but are unable to.” The 17 items were answered on a five-point Likert scale with 1 = not at all and 5 = always. The total score was obtained by summing up the scores of 17 items. The higher the score of MPAI was, the greater the level of mobile phone addiction was. This scale had satisfied reliability and validity (Huang et al., 2014). The Cronbach’s alpha was 0.870.

#### Revised Adult Attachment Scale (AAS)

The Chinese version (Du et al., 2015) of R-AAS (Collins, 1996) was adopted, which consisted of 18 items and assessed the attachment styles. Each item was scored on a five-point Likert scale with 1 = not at all like me and 5 = very much like me. Two attachment styles (anxiety and avoidance) were computed and followed Fraley and Spieker (2003) and Kaufman et al. (2019). The Attachment anxiety subscale consisted of six items and attachment avoidance consisted of 12 items. The overall scores of attachment anxiety and attachment avoidance were calculated by adding the 6 and 12 individual item scores, respectively (scores from reverse items were added after being computed as 6−*x*). Attachment anxiety was the extent to which people feared being abandoned and rejected, while attachment avoidance was the extent to which people were afraid of intimate relationships and avoided dependence on others (Kaufman et al., 2019). Insecure attachment refers to high scores of one or both AAS subscales, while secure attachment means low scores of both subscales. The Cronbach’s alphas for the attachment avoidance and attachment anxiety subscale were 0.674 and 0.816, respectively.

#### Interpersonal Relationship Comprehensive Diagnostic Scale (IRCDS)

This scale was compiled by Zheng Richang (Zheng, 1999) and used to assess the troubles or distresses of a person in interpersonal relationships. The IRCDS measures troubles or distresses in interpersonal conversation, making friends, association with different sex, and attitudes of dealing with people (Mou et al., 2019). There are 28 yes-or-no items in total, which includes items such as “You feel nervous on any social occasions” or “You feel unnatural when meeting a stranger.” The score of IRCDS was equal to the number of items answered with “Yes.” People with a high score on IRCDS are more likely to encounter interpersonal relationship issues. This scale has acceptable reliability and validity (Zheng, 1999). The Cronbach’s alpha coefficient was 0.879.

### Statistical Analysis

The SPSS and PROCESS (Hayes, 2012), a freely available computational macros for SPSS that integrated the mediation and moderation analysis, were used to analyze data. A *p*-value of 0.05 was considered statistically significant. Independent two-sample *t-*test was used to check for possible gender difference, urban–rural source difference, and singleton or non-singleton difference in mobile phone use, ACE, attachment styles, and interpersonal relationship. The Pearson correlation coefficient was conducted to test the strength of association between variables. Model 6 of the PROCESS template, which defined a serial multiple mediator model, was used to test the serial mediating role of attachment styles and interpersonal relationship. The macros provided the model of total effects and direct and indirect effects with bootstrap confidence interval based on 10,000 resamples.

## Results

The results of descriptive statistics for all variables are shown in Table 1. Results of skewness and kurtosis analysis revealed that the scores of mobile phone use, attachment anxiety, attachment avoidance, and interpersonal relationship were approximately normal distribution, while the score of ACE was positively skewed and leptokurtotic. Given the large sample size, the untransformed data were used in the following statistical analysis that followed Tabachnick and Fidell (2007).

**TABLE 1:** Descriptive statistics for study variables.

Measure	*M*	*SD*	Skewness	Kurtosis
Adverse childhood experience	0.81	1.479	2.680	9.092
Mobile phone addiction	42.73	11.107	0.232	–0.337
Interpersonal relationship	8.22	5.767	0.588	–0.186
Attachment avoidance	31.99	5.412	–0.074	0.847
Attachment anxiety	15.68	4.383	–0.040	–0.122

A previous study showed that there was a gender difference in network addiction (Aparicio-Martínez et al., 2020) and smartphone addiction (Chen et al., 2017). Independent two-sample *t*-test was employed to determine whether there are significant demographic differences in all variables. The results of *t*-test revealed that only-child students displayed significantly higher attachment avoidance scores than non-only-child students (*t* = 2.11, *p* = 0.035, Cohen’s *d* = 0.24, mean difference = 1.29). No other significant difference was found between gender difference, urban–rural source difference, and singletons or non-singletons in mobile phone use, ACE, attachment styles, and interpersonal relationship.

The results of Pearson correlation coefficients of ACE, mobile phone use, attachment styles, and interpersonal relationship are presented in Table 2. Results showed that the scores of ACE, attachment avoidance, attachment anxiety, interpersonal relationship, and mobile phone use were positively related to each other.

**TABLE 2:** Correlations between study variables.

Variables	1	2	3	4	5
1 Adverse childhood experience	–				
2 Attachment avoidance	0.252^∗∗∗^	–			
3 Attachment anxiety	0.275^∗∗∗^	0.490^∗∗∗^	–		
4 Interpersonal relationship	0.395^∗∗∗^	0.369^∗∗∗^	0.471^∗∗∗^	-	
5 Mobile phone addiction	0.211^∗∗∗^	0.203^∗∗∗^	0.409^∗∗∗^	0.397^∗∗∗^	–

*^∗∗∗^*p* < 0.001.*

The regression analysis and bootstrap method used followed Hayes (2013) to identify the serial multiple mediation effects of attachment style and interpersonal relationship between ACE and mobile phone use. The score of ACEQ-R was the independent variable and the score of MPAI was the dependent variable. The scores of R-AAS and IRCDS were mediating variables. To reduce type 1 errors caused by data distribution, the unstandardized regression coefficient was computed. The regression and bootstrapping method were used to calculate the significance of path coefficients and confidence interval for total, direct, and indirect effects. If the 95% bias-corrected bootstrap confidence intervals do not include zero, the mediation effects are regarded as significant (Hayes, 2013). The standardized regression coefficient was also computed using the standardized variables in the above model. To control the possible influences of demographic variables on smartphone addiction, the effects of covariates, including gender, age, major, urban or rural areas, and singletons or non-singletons, were controlled in all regression analysis following previous studies (Yuchang et al., 2017; Mei et al., 2018; Volkmer and Lermer, 2019). Additionally, attachment avoidance or anxiety was included simultaneously in the regression analysis as covariate, to control for one another.

**FIGURE 1:**
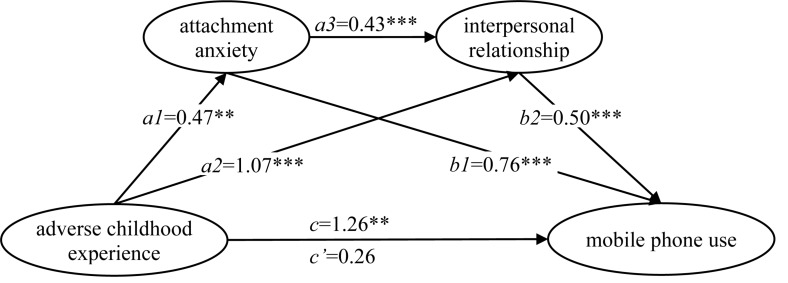
The serial multiple mediation of attachment anxiety and interpersonal relationship between adverse childhood experience and mobile phone use. Note: Path coefficients were shown in unstandardized regression coefficient. **p < 0.01, ***p < 0.001.

The results of serial multiple mediation of attachment anxiety and interpersonal relationship between ACE and mobile phone use are shown in Figure 1 and Table 3. The results showed that the total effect of ACE on mobile phone use was significant, while the direct effect was not statistically significant. In addition, there were three indirect effects that were significant based on the bootstrap confidence intervals: adverse childhood experience→attachment anxiety→mobile phone use, adverse childhood experience→interpersonal relationship→mobile phone use, and adverse childhood experience→attachment anxiety→interpersonal relationship→mobile phone use. The total indirect effect and three branch-indirect effects were 1.00, 0.36 (*a*1 × *b*1), 0.54 (*a*2 × *b*2), and 0.10 (*a*1 × *a*3 × *b*2), respectively. The ratio of total indirect effect and three branch-indirect effects to total effect were 79.40, 28.37, 42.84, and 8.19%, respectively.

**TABLE 3:** The serial multiple mediation of attachment anxiety and interpersonal relationship between adverse childhood experience and mobile phone use.

Path	Effect	SE	BootLLCI	BootULCI
Total effect (*c*)	0.21***	0.05	0.1033	0.3132
Direct effect (*c*′)	0.03	0.05	−0.0702	0.1393
*a1*	0.16**	0.05	0.0629	0.2530
*a2*	0.28***	0.05	0.1826	0.3680
*a3*	0.33***	0.05	0.2306	0.4364
*b1*	0.30***	0.06	0.1840	0.4186
*b2*	0.26***	0.06	0.1454	0.3764
**Indirect effects**				
Total indirect effects	0.13	0.03	0.0781	0.1970
Indirect 1	0.05	0.02	0.0191	0.0872
Indirect 2	0.01	0.01	0.0055	0.0280
Indirect 3	0.07	0.02	0.0337	0.1248

*N = 345, k = 10,000, ^∗∗^p < 0.01, ^∗∗∗^p < 0.001. Indirect 1, adverse childhood experience→attachment anxiety→mobile phone use; Indirect 2, adverse childhood experience→interpersonal relationship→mobile phone use; Indirect 3, adverse childhood experience→attachment anxiety-interpersonal relationship→mobile phone use. BootLLCI, bootstrapping lower limit confidence interval; BootULCI, bootstrapping upper limit confidence interval; SE, standard error; Effect, standardized regression coefficient.*

**FIGURE 2:**
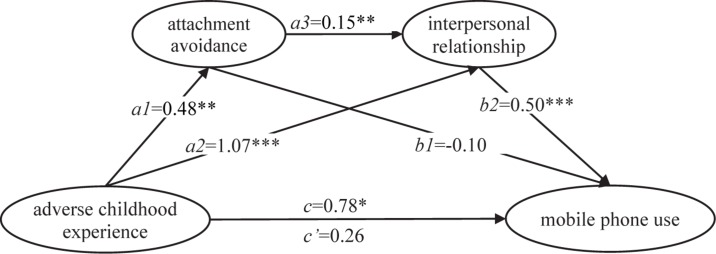
The serial multiple mediation of attachment avoidance and interpersonal relationship between adverse childhood experience and mobile phone use. Note: Path coefficients were shown in unstandardized regression coefficient. *p < 0.05, **p < 0.01, ***p < 0.001.

The results of serial multiple mediation of attachment avoidance and interpersonal relationship between ACE and mobile phone use are shown in Figure 2 and Table 4. The results showed that the total effect of ACE on mobile phone use was significant, while the direct effect was not statistically significant. In addition, there were two indirect effects that were significant based on the bootstrap confidence intervals: adverse childhood experience→interpersonal relationship→mobile phone use and adverse childhood experience→attachment avoidance→interpersonal relationship→mobile phone use. The total indirect effect and two branch-indirect effects were 0.53, 0.54 (*a*2 × *b*2) and 0.03 (*a*1 × *a*3 × *b*2), respectively. The ratios of total indirect effect and two branch-indirect effects to total effect were 66.93, 68.77, and 4.45%, respectively.

**TABLE 4:** The serial multiple mediation of attachment avoidance and interpersonal relationship between adverse childhood experience and mobile phone use.

Path	Effect	SE	BootLLCI	BootULCI
Total effect (*c*)	0.10^∗^	0.05	0.0031	0.2058
Direct effect (*c*′)	0.03	0.05	–0.0702	0.1393
*a1*	0.13^∗∗^	0.05	0.0353	0.2263
*a2*	0.28^∗∗∗^	0.05	0.1826	0.3680
*a3*	0.14^∗∗^	0.05	0.0333	0.2392
*b1*	–0.05	0.06	–0.1622	0.0618
*b2*	0.26^∗∗∗^	0.06	0.1454	0.3765
**Indirect effects**				
Total indirect effects	0.07	0.02	0.0289	0.1204
Indirect 1	–0.007	0.01	–0.0297	0.0065
Indirect 2	0.004	0.01	0.0008	0.0139
Indirect 3	0.07	0.02	0.0357	0.1263

*N = 345, k = 10,000, ^∗^p < 0.05, ^∗∗^p < 0.01, ^∗∗∗^p < 0.001. Indirect 1, adverse childhood experience→attachment avoidance→mobile phone use; Indirect 2, adverse childhood experience→interpersonal relationship→mobile phone use; Indirect 3, adverse childhood experience→attachment avoidance→interpersonal relationship-mobile phone use. BootLLCI, bootstrapping lower limit confidence interval; BootULCI, bootstrapping upper limit confidence interval; SE, standard error; Effect, standardized regression coefficient.*

## Discussion

The present research intended to test the indirect effect of earlier ACE on latter mobile phone addiction. Results showed that the serial multiple mediation of attachment style and interpersonal relationship was statistically significant between ACE and mobile phone use. This result supports the notion of attachment theory (Frederick and Goddard, 2008) that the earlier relationship between child and their caregiver was related to attachment disorder (Khantzian, 2011), such as addictive behavior (Parolin and Simonelli, 2016). It helped to explain how ACE results in smartphone addiction, mainly due to the serial mediation effects of attachment style and interpersonal relationship. In the following, these results would be discussed.

### The Correlation Between ACEs and Mobile Phone Addiction

Consistent with our assumption, ACE was found to be related to mobile phone addiction in Chinese college students. In accordance with this result, previous studies also revealed that ACEs were related to different kinds of addictive behaviors including alcohol abuse (Dube et al., 2002), food addiction (Holgerson et al., 2018), tobacco dependence (Walsh and Cawthon, 2014), and drug addiction (Gomez et al., 2018). Other epidemiological researches also revealed that earlier ACEs are linked to addictive behavior in adulthood (Dube et al., 2003; Anda et al., 2006). Recently, a review indicated that earlier ACEs resulted in long-lasting changes in dopamine, oxytocin, and glucocorticoid system, which might be related to addiction at molecular, neuroendocrine, and behavioral aspects (Kim et al., 2017). The present results further revealed that there was a close relationship between ACE exposure and phone addictive behaviors.

### The Mediation Effect of Attachment Anxiety

In line with our speculation, ACEs predicted mobile phone addiction directly and indirectly through attachment anxiety. The cognitive–behavioral model of Davis (2001) indicated that insecure attachment styles were a reliable predictor of some substance abuse. Addictive behavior was also regarded as attachment disorder (Parolin and Simonelli, 2016). Attachment theory indicated that people who have went through adverse experiences in early childhood would have difficulties in the communication with others in later life (Frederick and Goddard, 2008). A longitudinal study found that child abuse experiences were positively correlated with insecure attachment styles (Raby et al., 2017). Other studies also found similar association between abuse experiences and insecure attachment styles (Bakermans-Kranenburg and van Ijzendoorn, 2009; Corcoran and McNulty, 2018). Previous studies revealed that there was a close relationship between attachment anxiety and mobile phone addiction (Han et al., 2017; Yuchang et al., 2017; Kim and Koh, 2018). Shin et al. (2011) also found that attachment anxiety predicted alcohol use and problematic internet use. Thus, earlier ACEs might cause higher attachment anxiety. Then, the anxiety of being abandoned or rejected by other people might drive them to depend alternatively on smartphone for possible consolation, which might eventually lead to some level of mobile phone addiction. That is, the influence of ACEs on smartphone addiction in college students through the mediated effect of attachment anxiety.

### The Mediation Effect of Interpersonal Relationships

Additionally, ACEs also predicted mobile phone addiction directly and indirectly through interpersonal relationships. Emotional abuse during childhood induced more interpersonal problem with their parents, teachers, and peers (Lin et al., 2016). Other research found that earlier sexual abuse damaged the interpersonal functioning in a large sample of 2892 young adult women. A recent study revealed that earlier adversity positively related to the enhanced interpersonal difficulties in adulthood in a sample of 4006 (Poole et al., 2018). In addition, many studies showed that interpersonal relationships related closely to addiction behavior. For example, a study on Korean middle school students found that the problems of interpersonal relationships was correlated positively with internet addiction (Seo et al., 2009). A research on internet game players found that the internet game addiction was positively correlated with the quality of interpersonal relation with parents and peers (Kwon et al., 2011). A study on Chinese middle school students found that interpersonal relationships were significantly correlated with internet addiction disorder (Huang, 2009). Other studies also found that interpersonal relationships were related to Facebook addiction (Tang et al., 2016) and video game addiction (Choi et al., 2017). Thus, the earlier ACEs might cause lousy interpersonal relationships. Then, the problematic interpersonal relationships might drive them to depend on mobile phone for consolation, which might finally lead to mobile phone addiction.

### The Serial Multiple Mediation Model

The present serial multiple mediation analysis showed that earlier ACE indirectly influenced problematic mobile phone use through the effects of attachment styles and interpersonal relationship. Attachment theory (Frederick and Goddard, 2008) considered that the earlier relationship between child and caregiver was related to attachment disorder (Khantzian, 2011) such as addictive behavior (Parolin and Simonelli, 2016). The above discussions had indicated that ACEs were related to attachment style (Bakermans-Kranenburg and van Ijzendoorn, 2009; Raby et al., 2017; Corcoran and McNulty, 2018) and interpersonal relationships were linked to smartphone addiction (Huang, 2009; Seo et al., 2009; Kwon et al., 2011). A previous study also found that insecure attachment styles were positively related to interpersonal problems (Berry et al., 2006). Koelen et al. (2016) further found that there was a close association between attachment avoidance and interpersonal problems. Therefore, the earlier experience of neglect or abuse might induce the insecure attachment style like fear of being abandoned or rejected. Then, the insecure attachment style might cause interpersonal problems. Lastly, interpersonal problems might contribute to mobile phone addiction.

### Limitations

There are some limitations to the present research. Firstly, although the bootstrap method was used, the method of cross-sectional design might not clarify the causal associations between ACE and smartphone addiction and generate possible biased estimates of parameters (Maxwell and Cole, 2007). The pathways revealed in our serial multiple mediation model might need further longitudinal study. Secondly, the present research used a homogeneous sample that only involved college students. Further research should examine to what extent the present results can be verified in other groups, such as high school students and community groups. Thirdly, the present study only investigated the serial multiple mediation effects of attachment style and interpersonal relationship between ACE and problematic mobile phone use. Further research could investigate other potential influence factors that might influence mobile phone addiction, such as life stress (Chiu, 2014), alexithymia (Mei et al., 2018; Hao et al., 2019), and well-being (Volkmer and Lermer, 2019). Lastly, all measures and analyses were based on self-report data, which might overestimate the relations among variables because of shared method variance and biased recall (Glazier and Alden, 2017). It would be helpful to include parental and teacher reports of ACEs and mobile phone usage.

## Conclusion

The present research examined 345 Chinese college students and revealed that ACE, attachment anxiety, attachment avoidance, interpersonal relationship, and mobile phone addiction were positively related to each other. ACEs could predict mobile phone addiction indirectly through attachment style and interpersonal relationships. To better understand the addictive mobile phone usage, the college students who had experienced ACEs in early years should be subjected to special concern. Additionally, our research showed that the adjustment of insecure attachment style and interpersonal relationship would be more important in the treatment of addictive mobile phone usage in the future.

## Data Availability Statement

The datasets generated for this study are available on request to the corresponding author.

## Ethics Statement

All subjects signed the written informed consent form before they joined in the research, which was authorized by the Institutional Human Participants Review Board of Jining Medical University.

## Author Contributions

WL designed the study, analyzed the data, and wrote the manuscript. XZ and MC collected the data. GL revised the manuscript.

## Conflict of Interest

The authors declare that the research was conducted in the absence of any commercial or financial relationships that could be construed as a potential conflict of interest.
